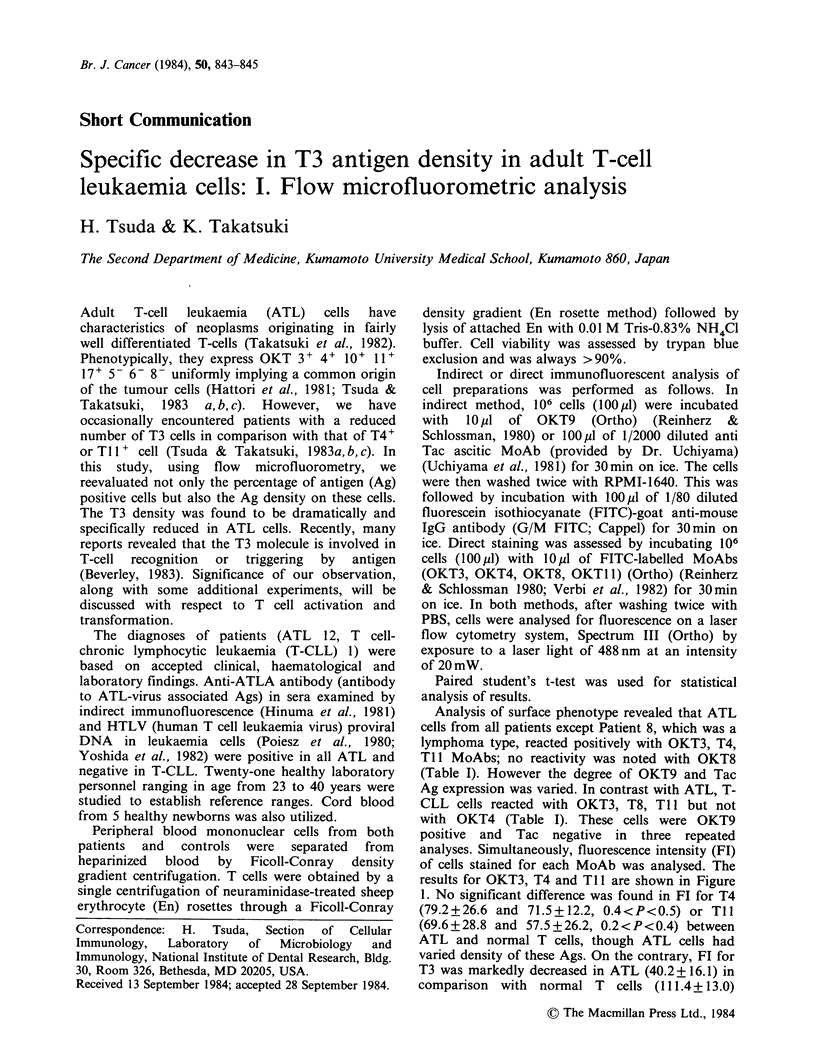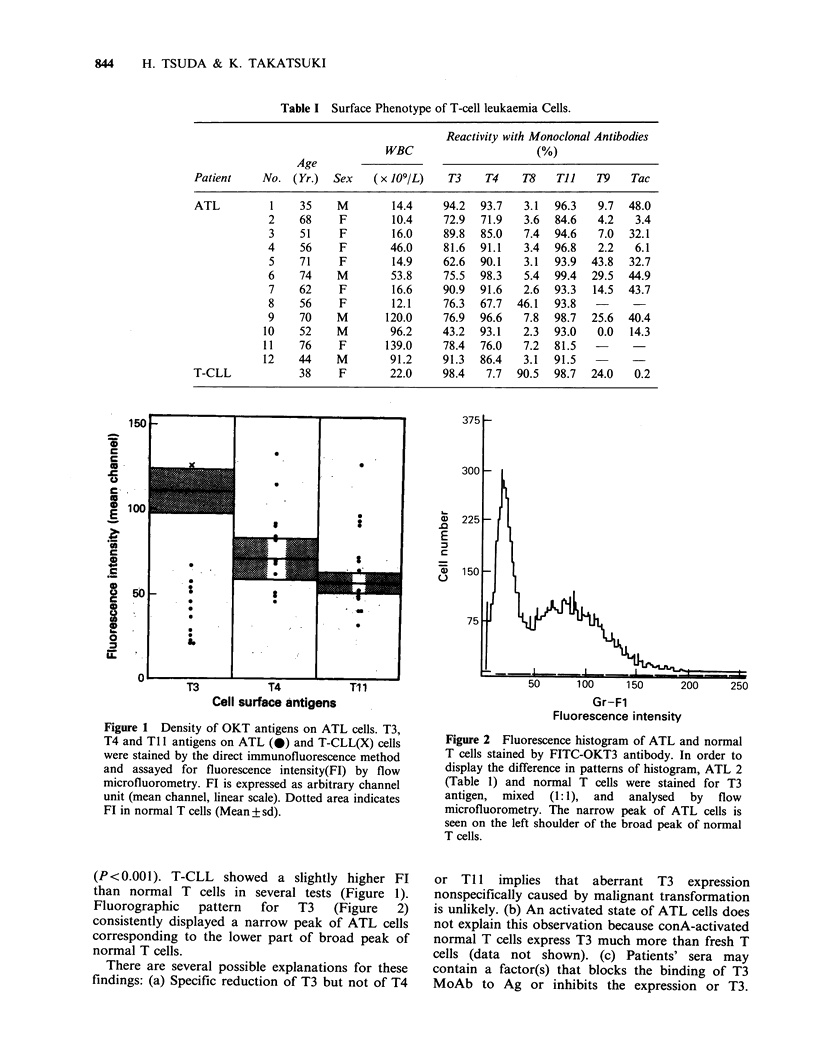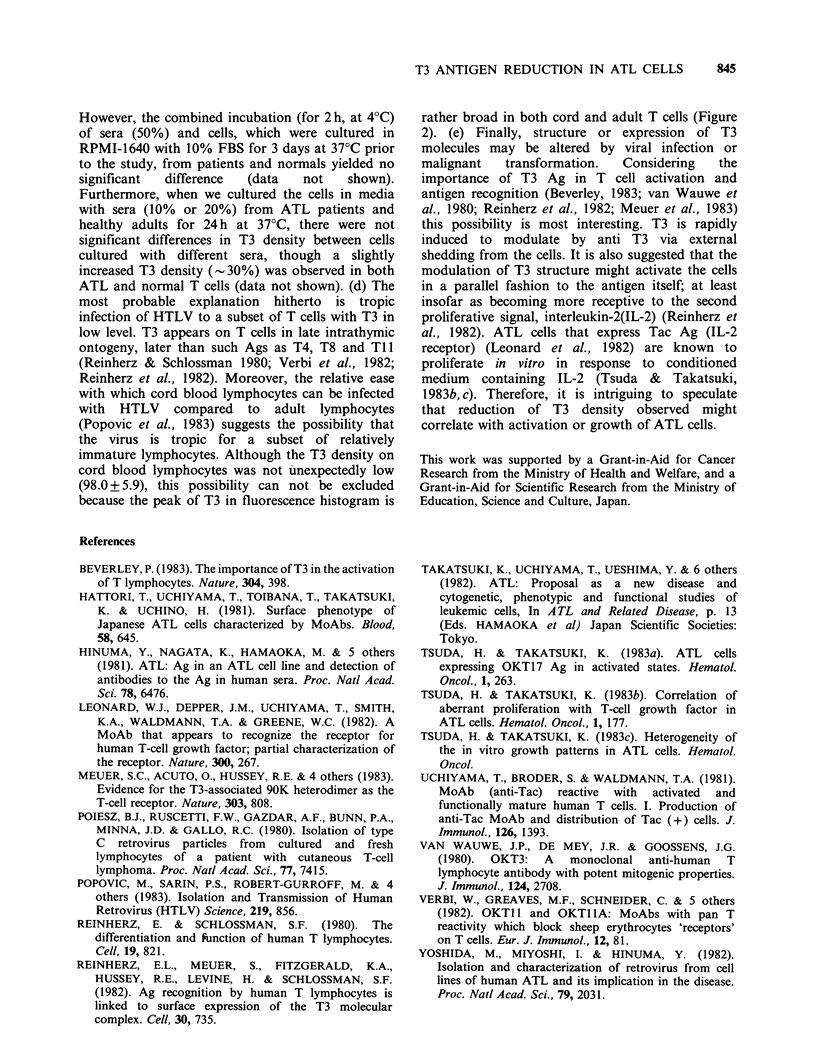# Specific decrease in T3 antigen density in adult T-cell leukaemia cells: I. Flow microfluorometric analysis.

**DOI:** 10.1038/bjc.1984.266

**Published:** 1984-12

**Authors:** H. Tsuda, K. Takatsuki


*Br. J. Cancer* (1984), **50**, 843–845

### Short Communication

### Specific decrease in T3 antigen density in adult T-cell leukaemia cells: I. Flow microfluorometric analysis

H. Tsuda & K. Takatsuki

*The Second Department of Medicine, Kumamoto University Medical School, Kumamoto 860, Japan*

Adult T-cell leukaemia (ATL) cells have characteristics of neoplasms originating in fairly well differentiated T-cells (Takatsuki *et al.*, 1982). Phenotypically, they express OKT $3^+$ $4^+$ $10^+$ $11^+$ $17^+$ $5^-$ $6^-$ $8^-$ uniformly implying a common origin of the tumour cells (Hattori *et al.*, 1981; Tsuda & Takatsuki, 1983 *a,b,c*). However, we have occasionally encountered patients with a reduced number of T3 cells in comparison with that of $T4^+$ or $T11^+$ cell (Tsuda & Takatsuki, 1983*a,b,c*). In this study, using flow microfluorometry, we reevaluated not only the percentage of antigen (Ag) positive cells but also the Ag density on these cells. The T3 density was found to be dramatically and specifically reduced in ATL cells. Recently, many reports revealed that the T3 molecule is involved in T-cell recognition or triggering by antigen (Beverley, 1983). Significance of our observation, along with some additional experiments, will be discussed with respect to T cell activation and transformation.

The diagnoses of patients (ATL 12, T cell-chronic lymphocytic leukaemia (T-CLL) 1) were based on accepted clinical, haematological and laboratory findings. Anti-ATLA antibody (antibody to ATL-virus associated Ags) in sera examined by indirect immunofluorescence (Hinuma *et al.*, 1981) and HTLV (human T cell leukaemia virus) proviral DNA in leukaemia cells (Poiesz *et al.*, 1980; Yoshida *et al.*, 1982) were positive in all ATL and negative in T-CLL. Twenty-one healthy laboratory personnel ranging in age from 23 to 40 years were studied to establish reference ranges. Cord blood from 5 healthy newborns was also utilized.

Peripheral blood mononuclear cells from both patients and controls were separated from heparinized blood by Ficoll-Conray density gradient centrifugation. T cells were obtained by a single centrifugation of neuraminidase-treated sheep erythrocyte (En) rosettes through a Ficoll-Conray density gradient (En rosette method) followed by lysis of attached En with 0.01 M Tris-0.83% $NH_4Cl$ buffer. Cell viability was assessed by trypan blue exclusion and was always >90%.

Indirect or direct immunofluorescent analysis of cell preparations was performed as follows. In indirect method, $10^6$ cells (100 $\mu$l) were incubated with 10 $\mu$l of OKT9 (Ortho) (Reinherz & Schlossman, 1980) or 100 $\mu$l of 1/2000 diluted anti Tac ascitic MoAb (provided by Dr. Uchiyama) (Uchiyama *et al.*, 1981) for 30 min on ice. The cells were then washed twice with RPMI-1640. This was followed by incubation with 100 $\mu$l of 1/80 diluted fluorescein isothiocyanate (FITC)-goat anti-mouse IgG antibody (G/M FITC; Cappel) for 30 min on ice. Direct staining was assessed by incubating $10^6$ cells (100 $\mu$l) with 10 $\mu$l of FITC-labelled MoAbs (OKT3, OKT4, OKT8, OKT11) (Ortho) (Reinherz & Schlossman 1980; Verbi *et al.*, 1982) for 30 min on ice. In both methods, after washing twice with PBS, cells were analysed for fluorescence on a laser flow cytometry system, Spectrum III (Ortho) by exposure to a laser light of 488 nm at an intensity of 20 mW.

Paired student's t-test was used for statistical analysis of results.

Analysis of surface phenotype revealed that ATL cells from all patients except Patient 8, which was a lymphoma type, reacted positively with OKT3, T4, T11 MoAbs; no reactivity was noted with OKT8 (Table I). However the degree of OKT9 and Tac Ag expression was varied. In contrast with ATL, T-CLL cells reacted with OKT3, T8, T11 but not with OKT4 (Table I). These cells were OKT9 positive and Tac negative in three repeated analyses. Simultaneously, fluorescence intensity (FI) of cells stained for each MoAb was analysed. The results for OKT3, T4 and T11 are shown in Figure 1. No significant difference was found in FI for T4 ($79.2 \pm 26.6$ and $71.5 \pm 12.2$, $0.4 < P < 0.5$) or T11 ($69.6 \pm 28.8$ and $57.5 \pm 26.2$, $0.2 < P < 0.4$) between ATL and normal T cells, though ATL cells had varied density of these Ags. On the contrary, FI for T3 was markedly decreased in ATL ($40.2 \pm 16.1$) in comparison with normal T cells ($111.4 \pm 13.0$)

Correspondence: H. Tsuda, Section of Cellular Immunology, Laboratory of Microbiology and Immunology, National Institute of Dental Research, Bldg. 30, Room 326, Bethesda, MD 20205, USA.
Received 13 September 1984; accepted 28 September 1984.

© The Macmillan Press Ltd., 1984

**Table I** Surface Phenotype of T-cell leukaemia Cells.

| *Patient* | *No.* | *Age (Yr.)* | *Sex* | *WBC* (×*10⁹/L*) | *Reactivity with Monoclonal Antibodies (%)* *T3* | *T4* | *T8* | *T11* | *T9* | *Tac* |
|---|---|---|---|---|---|---|---|---|---|---|
| ATL | 1 | 35 | M | 14.4 | 94.2 | 93.7 | 3.1 | 96.3 | 9.7 | 48.0 |
| | 2 | 68 | F | 10.4 | 72.9 | 71.9 | 3.6 | 84.6 | 4.2 | 3.4 |
| | 3 | 51 | F | 16.0 | 89.8 | 85.0 | 7.4 | 94.6 | 7.0 | 32.1 |
| | 4 | 56 | F | 46.0 | 81.6 | 91.1 | 3.4 | 96.8 | 2.2 | 6.1 |
| | 5 | 71 | F | 14.9 | 62.6 | 90.1 | 3.1 | 93.9 | 43.8 | 32.7 |
| | 6 | 74 | M | 53.8 | 75.5 | 98.3 | 5.4 | 99.4 | 29.5 | 44.9 |
| | 7 | 62 | F | 16.6 | 90.9 | 91.6 | 2.6 | 93.3 | 14.5 | 43.7 |
| | 8 | 56 | F | 12.1 | 76.3 | 67.7 | 46.1 | 93.8 | — | — |
| | 9 | 70 | M | 120.0 | 76.9 | 96.6 | 7.8 | 98.7 | 25.6 | 40.4 |
| | 10 | 52 | M | 96.2 | 43.2 | 93.1 | 2.3 | 93.0 | 0.0 | 14.3 |
| | 11 | 76 | F | 139.0 | 78.4 | 76.0 | 7.2 | 81.5 | — | — |
| | 12 | 44 | M | 91.2 | 91.3 | 86.4 | 3.1 | 91.5 | — | — |
| T-CLL | | 38 | F | 22.0 | 98.4 | 7.7 | 90.5 | 98.7 | 24.0 | 0.2 |

**Figure 1** Density of OKT antigens on ATL cells. T3, T4 and T11 antigens on ATL (●) and T-CLL(X) cells were stained by the direct immunofluorescence method and assayed for fluorescence intensity(FI) by flow microfluorometry. FI is expressed as arbitrary channel unit (mean channel, linear scale). Dotted area indicates FI in normal T cells (Mean ± sd).

**Figure 2** Fluorescence histogram of ATL and normal T cells stained by FITC-OKT3 antibody. In order to display the difference in patterns of histogram, ATL 2 (Table 1) and normal T cells were stained for T3 antigen, mixed (1:1), and analysed by flow microfluorometry. The narrow peak of ATL cells is seen on the left shoulder of the broad peak of normal T cells.

($P<0.001$). T-CLL showed a slightly higher FI than normal T cells in several tests (Figure 1). Fluorographic pattern for T3 (Figure 2) consistently displayed a narrow peak of ATL cells corresponding to the lower part of broad peak of normal T cells.

There are several possible explanations for these findings: (a) Specific reduction of T3 but not of T4 or T11 implies that aberrant T3 expression nonspecifically caused by malignant transformation is unlikely. (b) An activated state of ATL cells does not explain this observation because conA-activated normal T cells express T3 much more than fresh T cells (data not shown). (c) Patients' sera may contain a factor(s) that blocks the binding of T3 MoAb to Ag or inhibits the expression or T3.

However, the combined incubation (for 2 h, at 4°C) of sera (50%) and cells, which were cultured in RPMI-1640 with 10% FBS for 3 days at 37°C prior to the study, from patients and normals yielded no significant difference (data not shown). Furthermore, when we cultured the cells in media with sera (10% or 20%) from ATL patients and healthy adults for 24 h at 37°C, there were not significant differences in T3 density between cells cultured with different sera, though a slightly increased T3 density ($\sim$30%) was observed in both ATL and normal T cells (data not shown). (d) The most probable explanation hitherto is tropic infection of HTLV to a subset of T cells with T3 in low level. T3 appears on T cells in late intrathymic ontogeny, later than such Ags as T4, T8 and T11 (Reinherz & Schlossman 1980; Verbi *et al.*, 1982; Reinherz *et al.*, 1982). Moreover, the relative ease with which cord blood lymphocytes can be infected with HTLV compared to adult lymphocytes (Popovic *et al.*, 1983) suggests the possibility that the virus is tropic for a subset of relatively immature lymphocytes. Although the T3 density on cord blood lymphocytes was not unexpectedly low ($98.0 \pm 5.9$), this possibility can not be excluded because the peak of T3 in fluorescence histogram is rather broad in both cord and adult T cells (Figure 2). (e) Finally, structure or expression of T3 molecules may be altered by viral infection or malignant transformation. Considering the importance of T3 Ag in T cell activation and antigen recognition (Beverley, 1983; van Wauwe *et al.*, 1980; Reinherz *et al.*, 1982; Meuer *et al.*, 1983) this possibility is most interesting. T3 is rapidly induced to modulate by anti T3 via external shedding from the cells. It is also suggested that the modulation of T3 structure might activate the cells in a parallel fashion to the antigen itself; at least insofar as becoming more receptive to the second proliferative signal, interleukin-2(IL-2) (Reinherz *et al.*, 1982). ATL cells that express Tac Ag (IL-2 receptor) (Leonard *et al.*, 1982) are known to proliferate *in vitro* in response to conditioned medium containing IL-2 (Tsuda & Takatsuki, 1983*b, c*). Therefore, it is intriguing to speculate that reduction of T3 density observed might correlate with activation or growth of ATL cells.

This work was supported by a Grant-in-Aid for Cancer Research from the Ministry of Health and Welfare, and a Grant-in-Aid for Scientific Research from the Ministry of Education, Science and Culture, Japan.

### References

BEVERLEY, P. (1983). The importance of T3 in the activation of T lymphocytes. *Nature*, **304**, 398.

HATTORI, T., UCHIYAMA, T., TOIBANA, T., TAKATSUKI, K. & UCHINO, H. (1981). Surface phenotype of Japanese ATL cells characterized by MoAbs. *Blood*, **58**, 645.

HINUMA, Y., NAGATA, K., HAMAOKA, M. & 5 others (1981). ATL: Ag in an ATL cell line and detection of antibodies to the Ag in human sera. *Proc. Natl Acad. Sci.* **78**, 6476.

LEONARD, W.J., DEPPER, J.M., UCHIYAMA, T., SMITH, K.A., WALDMANN, T.A. & GREENE, W.C. (1982). A MoAb that appears to recognize the receptor for human T-cell growth factor; partial characterization of the receptor. *Nature*, **300**, 267.

MEUER, S.C., ACUTO, O., HUSSEY, R.E. & 4 others (1983). Evidence for the T3-associated 90K heterodimer as the T-cell receptor. *Nature*, **303**, 808.

POIESZ, B.J., RUSCETTI, F.W., GAZDAR, A.F., BUNN, P.A., MINNA, J.D. & GALLO, R.C. (1980). Isolation of type C retrovirus particles from cultured and fresh lymphocytes of a patient with cutaneous T-cell lymphoma. *Proc. Natl Acad. Sci.*, **77**, 7415.

POPOVIC, M., SARIN, P.S., ROBERT-GURROFF, M. & 4 others (1983). Isolation and Transmission of Human Retrovirus (HTLV) *Science*, **219**, 856.

REINHERZ, E. & SCHLOSSMAN, S.F. (1980). The differentiation and function of human T lymphocytes. *Cell*, **19**, 821.

REINHERZ, E.L., MEUER, S., FITZGERALD, K.A., HUSSEY, R.E., LEVINE, H. & SCHLOSSMAN, S.F. (1982). Ag recognition by human T lymphocytes is linked to surface expression of the T3 molecular complex. *Cell*, **30**, 735.

TAKATSUKI, K., UCHIYAMA, T., UESHIMA, Y. & 6 others (1982). ATL: Proposal as a new disease and cytogenetic, phenotypic and functional studies of leukemic cells, In *ATL and Related Disease*, p. 13 (Eds. HAMAOKA *et al*) Japan Scientific Societies: Tokyo.

TSUDA, H. & TAKATSUKI, K. (1983*a*). ATL cells expressing OKT17 Ag in activated states. *Hematol. Oncol.*, **1**, 263.

TSUDA, H. & TAKATSUKI, K. (1983*b*). Correlation of aberrant proliferation with T-cell growth factor in ATL cells. *Hematol. Oncol.*, **1**, 177.

TSUDA, H. & TAKATSUKI, K. (1983*c*). Heterogeneity of the in vitro growth patterns in ATL cells. *Hematol. Oncol.*

UCHIYAMA, T., BRODER, S. & WALDMANN, T.A. (1981). MoAb (anti-Tac) reactive with activated and functionally mature human T cells. I. Production of anti-Tac MoAb and distribution of Tac (+) cells. *J. Immunol.*, **126**, 1393.

VAN WAUWE, J.P., DE MEY, J.R. & GOOSSENS, J.G. (1980). OKT3: A monoclonal anti-human T lymphocyte antibody with potent mitogenic properties. *J. Immunol.*, **124**, 2708.

VERBI, W., GREAVES, M.F., SCHNEIDER, C. & 5 others (1982). OKT11 and OKT11A: MoAbs with pan T reactivity which block sheep erythrocytes 'receptors' on T cells. *Eur. J. Immunol.*, **12**, 81.

YOSHIDA, M., MIYOSHI, I. & HINUMA, Y. (1982). Isolation and characterization of retrovirus from cell lines of human ATL and its implication in the disease. *Proc. Natl Acad. Sci.*, **79**, 2031.